# Dual-mode varifocal Moiré metalens for quantitative phase and edge-enhanced imaging

**DOI:** 10.1515/nanoph-2025-0245

**Published:** 2025-08-06

**Authors:** Yi Lian, Yongqi Liu, Dewen Cheng, Cheng Chi, Yanjun Bao, Yongtian Wang

**Affiliations:** State Key Laboratory of Chips and Systems for Advanced Light Field Display, School of Optics and Photonics, 47833 Beijing Institute of Technology, Beijing 100081, China; Beijing Engineering Research Center of Mixed Reality and Advanced Display, School of Optics and Photonics, 47833 Beijing Institute of Technology, Beijing 100081, China; Guangdong Provincial Key Laboratory of Nanophotonic Manipulation, Institute of Nanophotonics, College of Physics and Optoelectronic Engineering, Jinan University, Guangzhou 511443, China

**Keywords:** quantitative phase imaging, transport-of-intensity equation, Moiré metalens, polarization-multiplexing, edge-enhanced imaging

## Abstract

Transport-of-intensity equation (TIE) as a noninterference method for quantitative phase imaging (QPI) has broad applications in micrographic imaging and optical metrology. Previous TIE-based QPI systems require the axial displacement of the detector to capture the axial intensity distributions, thus limiting the systems’ response speed, integration, and phase retrieval accuracy. Besides, the TIE-based phase imaging for edge positions with large phase gradients remains challenging. In this work, a compact polarization-multiplexed Moiré metalens is proposed to achieve QPI and edge-enhanced imaging for high-precision and unwrapping phase imaging. This Moiré metalens enables continuous zooming from 58.7 μm to 61.8 μm, allowing flexible selection of the detection positions. Under *x*-polarization light incidence, the metalens can achieve phase retrieval based on the TIE method, with the Root Mean Square Errors (RMSE) reaching 0.015 rad. Under *y*-polarization light incidence, the metalens realizes varifocal edge-enhanced imaging for amplitude and phase objects, with a minimum spatial resolution of 1.3 μm. This Moiré metalens opens a new avenue to develop compact, integrated, and multifunctional phase imaging devices and has potential applications in optical detection, microscopy, and biomedical imaging.

## 1 Introduction

Metasurfaces are artificially designed two-dimensional structures, composed of specially arranged subwavelength units [1]. The unique structure and arrangement of the meta-atoms endow metasurfaces with precise electromagnetic wave manipulation capabilities including phase regulation [2], [3], [4], polarization multiplexing [5], [6], [7], frequency selection [8], [9], *etc*. These capabilities broaden their application scopes in microscopic imaging [10], [11], [12], super-resolution imaging [13], [14], [15], vortex beam generation [16], [17], [18], [19], and so on [20], [21], [22], [23], [24], [25], [26], [27].

Varifocal metalenses have broad utilization in photographic equipment [28], micrography [29], [30], and augmented reality display [31], [32]. In the optical system, zooming typically requires axial displacement of several lens groups, resulting in bulky structures. Some novel zoom methods have been proposed. The Alvarez metalens realizes zoom via lateral displacement of two specially designed metasurfaces [33], [34]. Tunable metalenses based on liquid crystal or microelectromechanical elements achieve zoom through electrical or thermal excitation mechanism [35], [36]. Moreover, deformable lenses using liquid or soft elastic materials have also been developed [37]. However, these zoom methods exhibit high structural complexity. Recently, the concept of varifocal Moiré metalens has been proposed. The Moiré metalens consists of two axially aligned metasurfaces, whose superposition phase profiles satisfy the focusing distributions [38]. The zoom functionality can be realized by rotating the metasurfaces relative to each other.

Brightfield imaging has limited sensitivity for weakly absorbing or transparent samples detection. Quantitative phase imaging (QPI) is a label-free imaging method, which has been widely used in microscopic imaging and optical metrology [39]. QPI measures the sample-induced phase delay through techniques including holography, tomographic imaging, and transport of intensity equation (TIE) [40], [41], [42], [43]. TIE is a noninterferometric phase retrieval method based on the transport of intensity equation. In comparison with other QPI methods, TIE exhibits lower computational complexity and requires no reference light or phase unwrapping. Moreover, it is compatible with commercially available microscopy setups employing Köhler illumination [44]. TIE is a second-order elliptic partial differential equation that relates the axial derivative of intensity to the transverse phase distribution. This method plays a significant role in microscope, biomedical imaging, and optical detection [45], [46], [47], [48], [49], [50]. Metasurface-enabled phase retrieval based on TIE has attracted significant research attention. Engay et al. proposed a polarization-dependent metasurface that can simultaneously record the axial intensity profiles for TIE [51]. This metasurface captures only two axial intensity images, which limits the phase retrieval accuracy. Min et al. proposed a metalens whose zoom function is achieved by polarization rotation of the incident light [52]. This approach employed the varifocal metalens to replace mechanical movement for acquiring the axial intensity images. However, chiral samples demonstrate optical anisotropy to polarized light. Variations in incident light’s polarization state during the zoom operation cause imaging variation, thus affecting the phase retrieval accuracy. Wang et al. proposed a wavelength-dependent varifocal metalens for TIE-based QPI [53]. They utilized the dispersive characteristic of the metalens to realize spectral focal shifting for acquisition of the defocus images. These two studies both introduced varifocal metalens to acquire the axial intensity images, so the system’s compactness can be improved. Nevertheless, the zooming function of these meta-devices is achieved by rotating the polarizer or changing the color filter, which can be further improved.

In this work, we proposed a dual-mode varifocal Moiré metalens for QPI and edge-enhanced imaging. This metalens consists of two axially aligned metasurfaces and exhibits focusing and vortex-focusing phase profiles, respectively, in the *x*- and *y*-polarization states. Under *x*-polarization light incidence, the metalens achieves continuous zoom from 58.7 μm to 61.8 μm to capture multiple axial intensity images. Combined with the TIE algorithm, the metalens can achieve a high-precision phase retrieval with a Root Mean Square Errors (RMSE) of 0.015 rad. Additionally, under *y*-polarization light incidence, the Moiré metalens can achieve varifocal edge-enhanced imaging for both amplitude and phase objects, with a minimum spatial resolution of 1.3 μm.

## 2 Theory and method

### 2.1 Principle of the phase imaging based on Moiré metalens

**Figure 1:**
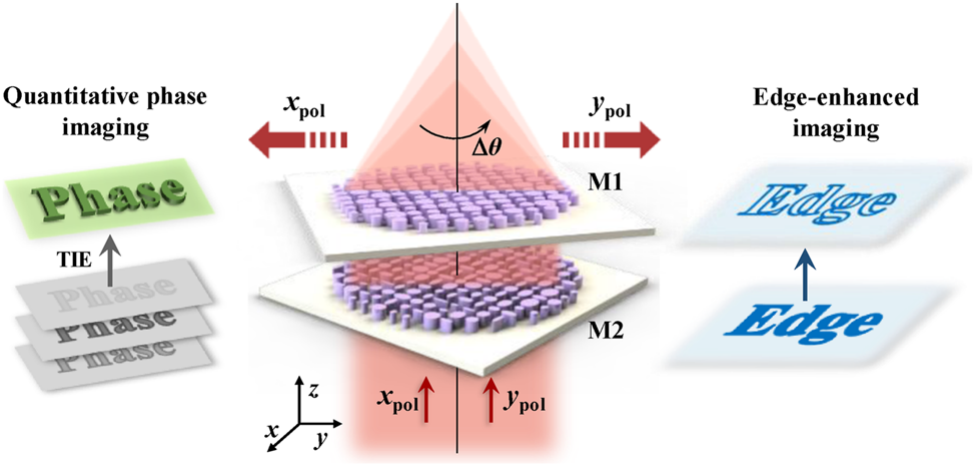
Schematics of the polarization-multiplexed Moiré metalens for quantitative phase imaging (left panel) and edge-enhanced imaging (right panel). The focal length varies continuously as the relative angle Δ*θ* changes. The metalens enables the acquisition of axial intensity profiles for QPI in the *x*-polarization state and varifocal edge-enhanced imaging in the *y*-polarization state.

Here, we proposed a dual-mode varifocal Moiré metalens for QPI and edge-enhanced imaging, as shown in Figure 1. This Moiré metalens is composed of two axially aligned metasurfaces M1 and M2. The metalens enables continuous optical zoom by rotating the metasurface M1. This varifocal metalens can obtain the required axial intensity images under *x*-polarization light incidence, with all metasurfaces maintain the axial position fixed. Combined with the TIE algorithm, the Moiré metalens can achieve high-precision phase retrieval. Additionally, this metalens generates a vortex-focusing phase profile under *y*-polarization light incidence. Therefore, edge-enhanced imaging can be achieved by convolving image with the point spread function (PSF) of the metalens as $U_{\text{out}}\left(x,y\right)=U_{\text{in}}\left(x,y\right)⊗P\left(r,\varphi \right)$ . Here, ⊗ is the convolution symbol, *U*_out_ and *U*_in_ refer to the output and input light field, respectively, $\left(x,y\right)$ are the spatial coordinates of the sampling plane, and $\left(r,\varphi \right)$ are the polar coordinates. The $P\left(r,\varphi \right)=F\left[\exp\left(i\varphi \right)\right]$ corresponds to the vortex phase distribution, and here $F\left[\cdot \right]$ refers to the Fourier transform. The vortex phase metasurface can perform a two-dimensional spatial differentiation on the input light field.

The quantitative relationship between the axial intensity derivative and the transverse phase distribution can be described through a second-order elliptic partial differential equation [54]:

(1) $$-k\frac{\partial I\left(r,z\right)}{\partial z}=\nabla \cdot \left[I\left(r,z\right)\nabla \phi \left(r\right)\right]$$

where *k* refers to the wavenumber 2*π*/*λ* and the operating wavelength *λ* is 633 nm, $r=\left(x,y\right)$ are the transverse coordinates, $I\left(r,z\right)$ is the intensity distribution on the recorded plane at the propagation distance *z*, $\phi \left(r\right)$ refers to the phase distribution on the recorded plane, and ∇ is the two-dimensional gradient operator. For pure phase object (*I* ≈ constant), Eq. (1) can be simplified as:

(2) $$-k\frac{\partial I\left(r,z\right)}{\partial z}=I\nabla^{2}\phi \left(r\right)$$

Hence, the phase distribution $\phi \left(r\right)$ can be derived from the axial intensity derivative ∂*I*/∂*z*. Theoretically, phase retrieval requires only two intensity images at different propagation distances. However, in order to suppress noise in phase retrieval, multiple intensity profiles are typically required. Previous TIE systems generally capture the required intensity images by axially moving the detector. However, this approach limits systems’ compactness and response speed. Additionally, the impact of mechanical movement errors on retrieval accuracy is non-negligible. We obtained the required axial intensity images via the varifocal Moiré metalens zooming from 58.7 μm to 61.8 μm, corresponding to 3.1 μm defocus range.

### 2.2 Design and features of the Moiré metalens

The specially designed metasurfaces enable the metalens to exhibit Moiré and vortex-focusing phase profiles, respectively, in the *x*- and *y*-polarization states. The Moiré phase *ϕ*_*f*_ of the metalens can be expressed as:

(3) $$\phi_{f}\left(r\right)=-\frac{\pi r^{2}}{\lambda f}$$

The vortex phase profile *ϕ*_*e*_ is written as:

(4) $$\phi_{e}\left(r,\varphi \right)=-\frac{\pi r^{2}}{\lambda f}+\varphi$$

where *f* refers to the focal length, and $\left(r,\varphi \right)$ are the polar coordinates of the metalens. The phase profile of the polarization-dependent metasurface M2 is:

(5) $$\phi_{\text{M}2,x}=-\text{round}\left(\frac{\pi r^{2}}{\lambda f}\left(\varphi +\theta_{2}\right)\right)$$

(6) $$\phi_{\text{M}2,y}=-\text{round}\left(\frac{\pi r^{2}}{\lambda f}\left(\varphi +\theta_{2}\right)\right)+\varphi +\theta_{2}$$

Here, *θ*_2_ is the rotation angle of M2, *ϕ*_M2,*x*_ and *ϕ*_M2,*y*_ refer to the phase distributions, respectively, in the *x*- and *y*-polarization states. $\text{round}\left(\cdot \right)$ is the integer operator; thus, the discontinuity at the polar angle *φ* = *π* can be removed [55]. Additionally, the phase distribution of the polarization-independent metasurface M1 can be expressed as:

(7) $$\phi_{\text{M}1}=\text{round}\left(\frac{\pi r^{2}}{\lambda f}\left(\varphi +\theta_{1}\right)\right)$$

where *θ*_1_ refers to the rotation angle of M1. The phase profiles of the Moiré metalens can be written as the superposition of M1 and M2 phase distributions:

(8) $$\left\{\begin{array}{c} \phi_{x}=-\text{round}\left(\frac{\pi r^{2}}{\lambda f}\Delta \theta \right)\;\\ \phi_{y}=-\text{round}\left(\frac{\pi r^{2}}{\lambda f}\Delta \theta \right)+\varphi +\theta_{2}\; \end{array}\right.$$

where *ϕ*_*x*_ and *ϕ*_*y*_ represent the phase profiles of the Moiré metalens, respectively, under *x*- and *y*-polarization light incidence, and Δ*θ* = *θ*_2_ − *θ*_1_ refers to the relative angle between the two metasurfaces. Therefore, the relationship between the actual focal length *f*_*θ*_ and the relative angle Δ*θ* can be expressed as:

(9) $$f_{\theta }=\frac{f}{\Delta \theta }$$

Hence, the Moiré metalens enables flexible and continuous focal length adjustment via changing the relative angle Δ*θ*.

**Figure 2:**
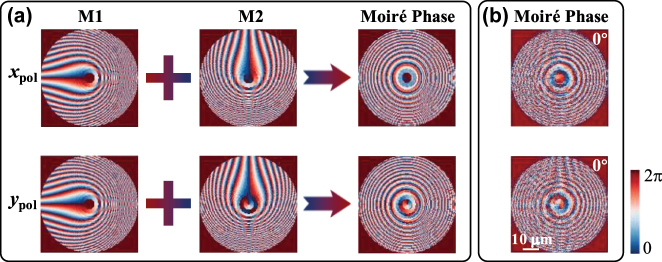
Phase distributions of the polarization-multiplexed Moiré metalens. (a) Theoretical phase distributions of metasurfaces M1, M2 and the superimposed Moiré phase profiles of the Moiré metalens under *x*-polarized and *y*-polarized light incidence, respectively. (b) Simulated phase distributions of the Moiré metalens under *x*-polarized and *y*-polarized light incidence, respectively.

The theoretical phase profiles of the Moiré metalens are demonstrated in Figure 2a. It’s illustrated that the phase distribution of metasurface M1 remains unchanged under different polarization states, corresponding to Eq. (7). In the *y*-polarization state, metasurface M2 introduces an additional vortex phase compared to the *x*-polarization state, as expressed in Eqs. (5) and (6). The metalens exhibits the Moiré and vortex phase profiles, respectively, under the two orthogonal polarization states, as shown in Eq. (8). Close agreement is achieved between theoretical and simulated phase distributions of the Moiré metalens, as illustrated in Figure 2a and b.

**Figure 3:**
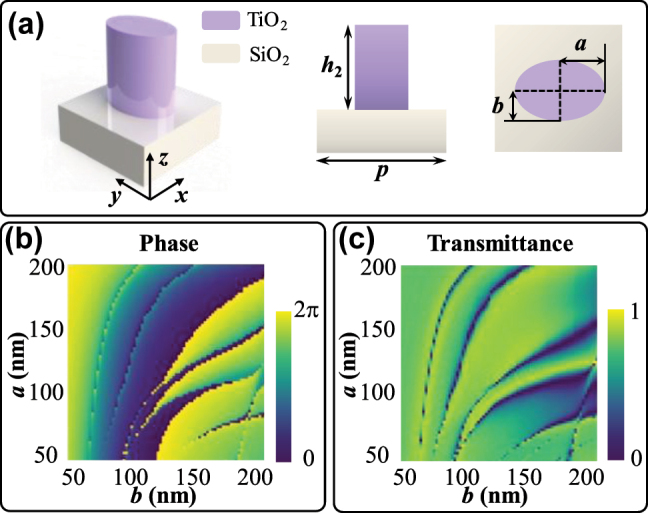
Schematics and characteristics of the meta-atoms. (a) Three-dimensional view, side view, and top view of the meta-atom. (b) The phase distribution chart corresponding to different elliptic parameters *a* and *b* of the nanopillars under *x*-polarization light incidence. (c) The transmittance distribution chart corresponding to different elliptic parameters *a* and *b* of the nanopillars under *x*-polarization light incidence.

High refractive index material TiO_2_ (*n* = 2.58) is used for the metalens structure, with SiO_2_ (*n* = 1.45) as the substrate. In the bilayer metasurfaces, M1 is composed of the nanocylinders, while M2 consists of the elliptical nanopillars. In order to endow the metalens with polarization-multiplexing capability for dual-mode imaging, we construct M2 with the polarization-sensitive units. We compose M1 with the polarization-independent units; thus, the superposition phase of M1 and M2 can exhibit the corresponding diverse phase profiles in different polarization states, as shown in Eq. (8). Besides, the phase distribution of M1 can remain constant during rotation. The structure of the elliptical nanopillar in M2 is shown in Figure 3a, where the periodicity of the meta-atoms is *p* = 450 nm, the height of the nanopillars is *h*_2_ = 600 nm. The long and short semi-axes of the ellipse *a* and *b* vary from 50 to 200 nm, respectively. The highest aspect ratio of the TiO_2_ is 1:6, satisfying the fabrication constraints [56]. Considering the fabrication tolerances of the elliptical cylinder structures, we verify the feasibility of the design through simulation. The parameter sweep was implemented through the commercial software FDTD (Ansys Lumerical FDTD). In the simulation, the periodic boundary condition is set in the *x* and *y* direction, while the perfectly matched layer (PML) boundary condition is set in the *z* direction. The mesh size is set as 10 nm. As *h*_2_ is close to the working wavelength *λ* = 633 nm, the meta-atoms can act as truncated waveguides and thus feature enhanced phase control capability. The proposed metalens can also be tuned to the infrared wavelength range. For an infrared-band device, crystalline silicon (c-Si) can serve as a constituent material for the metasurface, which can be compatible with semiconductor processing [57]. The phase distributions of the meta-atoms cover 0 to 2*π*, satisfying the needs of the metalens design, as shown in Figure 3b. As for the meta-atoms of M1, the height of the nanocylinders is *h*_1_ = 650 nm with the radius *r* ranging from 50 nm to 120 nm, and the corresponding phase distributions also satisfy the 2*π* phase range. We select the meta-atoms covering the full 2*π* phase range with the transmittance higher than 80 % to construct the Moiré metalens, as illustrated in Figure 3c. The maximum error between the real and target phase is 0.4 rad.

**Figure 4:**
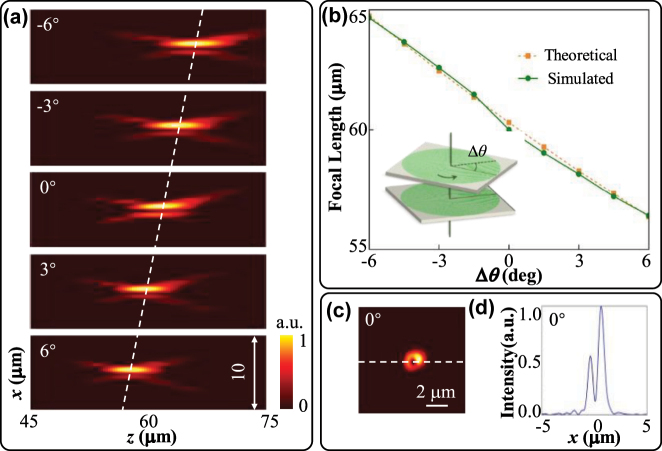
Simulated results of the Moiré metalens in the *y*-polarization state. (a) Normalized intensity distributions on the *x*–*z* plane corresponding to different relative angles Δ*θ*. (b) Theoretical and simulated focal length *f*_*θ*_ variation curves under different relative angles Δ*θ*. Normalized intensity distribution on the focal plane (c) and *x*-direction tangent (d) at the relative angle Δ*θ* of 0°.

To verify the structural design, a Moiré metalens with an aperture of 58 μm is constructed in the commercial software FDTD. The simulation area is set to a three-dimension domain of 58 × 58 × 5 μm^3^, and the mesh size is set as 10 nm. We set the PML boundary conditions in the *x*, *y*, and *z* directions. The two axially aligned metasurfaces M1, M2 constitute the compact metalens, with an axial distance of 200 nm. The focusing effect can be calculated based on Fresnel diffraction theory. The simulated intensity distributions in the *x*–*z* plane and focal plane under *y*-polarization light incidence are demonstrated in Figure 4. The corresponding focal length *f*_*θ*_ increases from 56.4 μm to 64.7 μm with the relative angle Δ*θ* decreasing from 6° to −6°, as demonstrated in Figure 4a and b. The measured relationship between *f*_*θ*_ and Δ*θ* shows excellent agreement with the theoretical function, and the maximum deviation doesn’t exceed 0.23 μm. The slight discrepancy between the theoretical and simulated focal length *f*_*θ*_ stems from phase matching error. The periodic boundary condition is applied in the simulation of a single meta-atom. However, in the simulation of the entire metalens, the adjacent meta-atoms may not be identical. The mutual interaction between meta-atoms can cause a local deviation in the Moiré phase, resulting in a small random shift in the focal length. Due to the inclusion of vortex phase, the intensity profile in the focal plane shows a donut-shaped distribution, as shown in Figure 4c and d.

**Figure 5:**
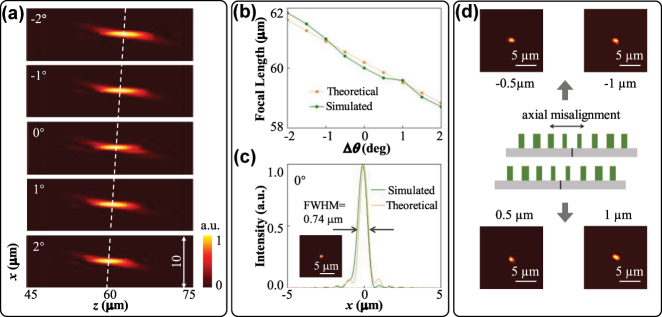
Simulated results of the Moiré metalens in the *x*-polarization state. (a) Normalized intensity distributions on the *x*–*z* plane corresponding to different relative angles Δ*θ*. (b) Theoretical and simulated focal length *f*_*θ*_ variation curves under different relative angles Δ*θ*. (c) The FWHM of the metalens at the relative angle Δ*θ* of 0°, the inset illustrates the focal spot profile. (d) Diagrams of the metasurfaces misalignment and focal spot maps under different axial misalignment distances at the relative angle Δ*θ* of 0°.

In the *x*-polarization state, the Moiré metalens realizes continuous zoom. As illustrated in Figure 5a and b, the focal length *f*_*θ*_ increases by 3.1 μm (58.7 μm–61.8 μm) with the relative angle Δ*θ* decreasing by 4° (2° to −2°), matching the theoretical calculations. Figure 5c depicts the full width at half maximum (FWHM) of the metalens at the relative angle Δ*θ* of 0°. The measured FWHM is 0.74 μm, which is close to the diffraction limit of *λ*/2NA. The simulated results prove that the designed Moiré metalens can achieve flexible and continuous adjustment of the focal length *f*_*θ*_ by regulating the relative angle Δ*θ*. Moreover, we also simulated potential axial misalignments during the experiment by slightly offsetting the two metasurfaces M1 and M2. The different focal spots under different axial misalignment distances are demonstrated in Figure 5d, and within the 2 µm (−1 µm–1 µm) offset range, the metalens can maintain consistent focusing performance. In practical application, it’s necessary to maintain the two metasurfaces center-aligned. However, the impact of minor axial misalignment on focusing performance of the proposed Moiré metalens is controllable.

## 3 Results and discussion

### 3.1 Quantitative phase imaging performance

**Figure 6:**
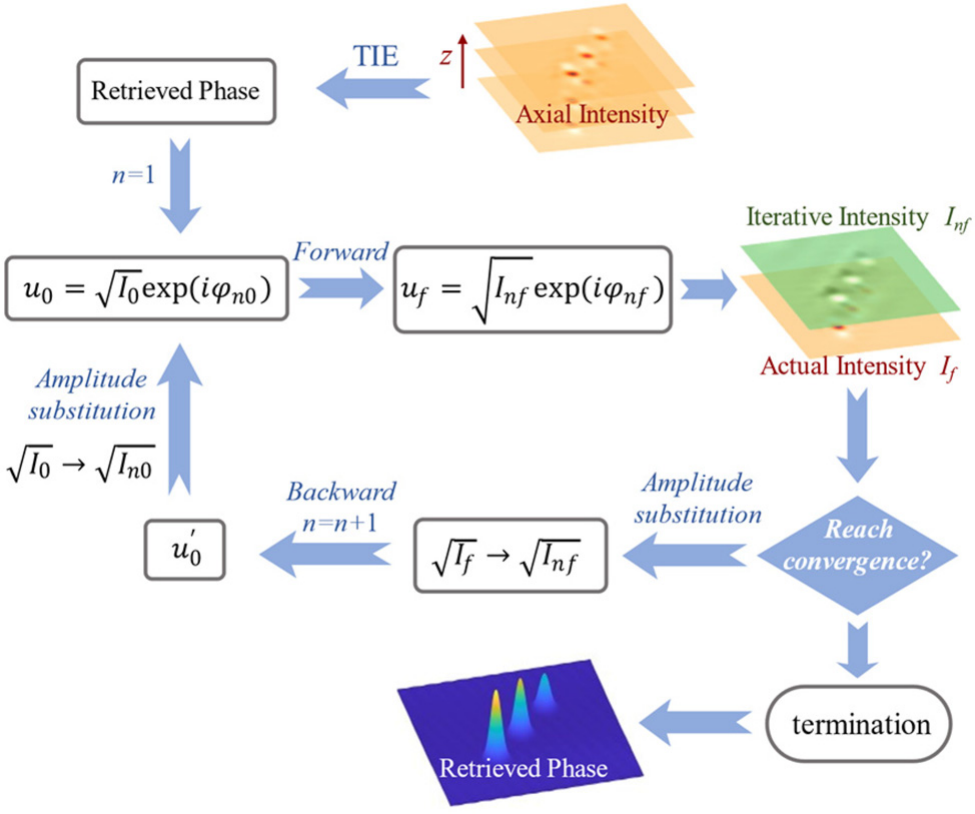
Flowchart of the phase retrieval method.

We combine iterative angular spectrum and the TIE method (AS-TIE method) to achieve high-precision QPI. In comparison with other phase retrieval methods, AS-TIE has advantages of low computational complexity and phase-unwrapping-free. The flowchart is demonstrated in Figure 6.

First, we extract the axial intensity derivative for phase retrieval, based on the zoom capability of the Moiré metalens. The selection of the defocus distances has a significant impact on the results’ accuracy. Too short defocus distance can’t filter the noise, whereas too long defocus distance will lead to the loss of high-frequency information. The phase profile can be retrieved from unequally spaced intensity distributions. The axial intensity derivative is represented by a linear combination of the intensity distributions at different defocus distances. The extraction of the axial intensity derivative ∂*I*/∂*z* is based on the higher-order Taylor expansion of the measured intensity. The calculations of ∂*I*/∂*z* and the reconstructed phase $\phi \left(r\right)$ are written as [58]:

(10) $$\left\{\begin{array}{c} \frac{\partial I}{\partial z}=\overset{N}{\underset{n=1}{\sum }}C_{n}\cdot \left[I_{zn}\left(r\right)-I_{0}\left(r\right)\right]\;\\ C_{n}=\frac{\overset{N}{\underset{\begin{array}{c} i=1\\ i\neq n \end{array}}{\prod }}z_{i}}{z_{n}\cdot \overset{N}{\underset{\begin{array}{c} i=1\\ i\neq n \end{array}}{\prod }}\left(z_{i}-z_{n}\right)}\;\\ \phi \left(r\right)=-\frac{k}{I}\nabla^{-2}\left(\frac{\partial I}{\partial z}\right)\; \end{array}\right.$$

**Figure 7:**
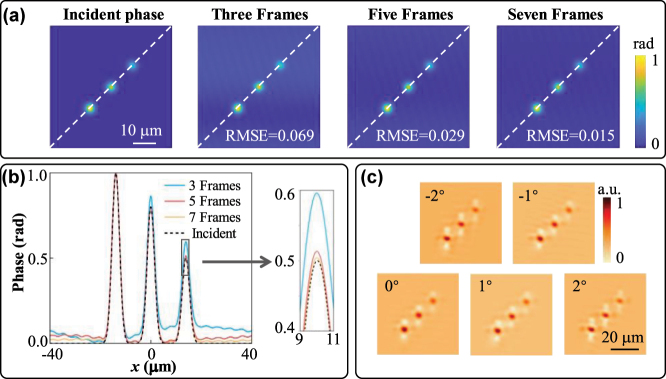
Quantitative phase imaging of the Moiré metalens. (a) The incident phase profile and the reconstructed phase profiles from different frames. The input image exhibits uniform intensity (*I* = 1) and the phase satisfies the three-peak Gauss distribution with different peak intensities. (b) Comparison of the target profile and the reconstructed phase profiles from different frames. (c) The intensity distributions of different relative angles Δ*θ*.

Here, *n* is the serial number of the defocus plane, $I_{zn}\left(r\right)$ refers to the intensity distribution of the *n*-th defocus plane, and $I_{0}\left(r\right)$ is the intensity distribution of the in-focus plane. *C*_*n*_ is the corresponding factor, and *z*_*n*_ refers to the defocus distance of the *n*-th defocus plane. The intensity distributions corresponding to different axial distances *z*_*n*_ can be obtained based on the Moiré metalens, as demonstrated in Figure 7c. The axial intensity derivative ∂*I*/∂*z* can be obtained based on Eq. (10). According to Eq. (2), the phase profile can be obtained by solving the Poisson equation, as shown in Eq. (10). The calculation can be simplified in Fourier domain, and the Laplace operator is $\nabla^{-2}=-1/\left[4\pi^{2}\left(f_{x}^{2}+f_{x}^{2}\right)\right]$ , where *f*_*x*_ and *f*_*x*_ are the spatial frequency coordinates.

Second, the previously obtained phase profile will be input as the initial value into the iterative angular spectrum algorithm. According to the angular spectrum theory, the forward and backward propagation process between the object and image plane (the focal plane) can be represented by the angular spectral transfer function *H*. The light field relation can be expressed as:

(11) $$u_{i}\left(x_{i},y_{i}\right)=F^{-1}\left\{F\left\{u_{0}\left(x_{0},y_{0}\right)\cdot H\right\}\right\}$$

where *u*_0_ and *u*_*i*_ refer to the optical field distribution of the object and image plane, respectively. The initial incident complex amplitude in this iteration is:

(12) $$u_{0}=\sqrt{I_{0}}\exp\left(i\varphi_{0}\right)$$

Here, *φ*_0_ is the previously extracted phase profile, and *I*_0_ refers to the intensity distribution of the incident plane. As for pure phase object, the incident intensity distribution is uniform, *i.e.*, *I*_0_ ≈ 1. The light field distribution of the focal plane can be calculated based on the forward propagation Eq. (11). According to the calculated focal plane complex amplitude $u_{f}=\sqrt{I_{nf}}\exp\left(i\varphi_{nf}\right)$ , the corresponding focal intensity distribution *I*_*nf*_ of the *n*-th cycle can be obtained. The iteration value *I*_*nf*_ should be compared to the measured focal intensity *I*_*f*_, and $\left|I_{f}-I_{nf}\right|$ is taken as the iterative evaluation function. If the evaluation value satisfies the criteria, the iteration terminates; otherwise, the iteration continues. Then, the focal amplitude $\sqrt{I_{nf}}$ should be replaced by the measured value $\sqrt{I_{f}}$ . Furthermore, the derived incident complex amplitude $u_{n0}=\sqrt{I_{n0}}\exp\left(i\varphi_{n0}\right)$ is obtained based on backward propagation. Moreover, we perform the amplitude substitution again, replacing $\sqrt{I_{n0}}$ with the actual value $\sqrt{I_{0}}$ . Repeat the process until the iteration converges or the maximum iteration count is met.

To test the phase retrieval accuracy, we choose the phase profile containing three different-peak Gauss distributions as the input phase profile, as illustrated in Figure 7a. We select this target phase to verify the proposed meta-device’s capability for phase reconstruction across the varying peak intensities and spatial coordinates. The system’s focal length is defined as 60 μm of 0° relative angle. As demonstrated in Figure 7a and b, the proposed Moiré metalens can achieve a high-precision phase retrieval. The reconstructed phase based on three images shows a larger gap from the target phase, and the background noise introduces notable interference. The retrieval accuracy can be improved by increasing the number of sample images. It can be observed that the reconstructed phase distributions with 5 and 7 frames closely match the target. The RMSE reach 0.029 rad and 0.015 rad, corresponding to the wavefront errors of 0.0046*λ* and 0.0024*λ*, respectively, and the calculation formula of RMSE is:

(13) $$\text{RMSE}=\sqrt{\frac{1}{N^{2}}\overset{N}{\underset{i=1}{\sum }}\overset{N}{\underset{j=1}{\sum }}{\left(\varphi_{o}\left(i,j\right)-\varphi_{r}\left(i,j\right)\right)}^{2}}$$

where $\varphi_{o}\left(i,j\right)$ and $\varphi_{r}\left(i,j\right)$ refer to the input and reconstructed phase, respectively, of each pixel, and *N* is the pixel number. As mentioned above, except for the intensity image frames, the defocus distance also has a significant impact on the phase retrieval accuracy. To simplify the operation and calculation process, the five-frame phase retrieval is a better choice, and the corresponding imaging distances are −1.3 μm, −0.4 μm, 0 μm, 1.0 μm, and 1.8 μm, corresponding to the rotation angles of 2°, 1°, 0°, −1°, and −2°.

### 3.2 Edge-enhanced imaging performance

**Figure 8:**
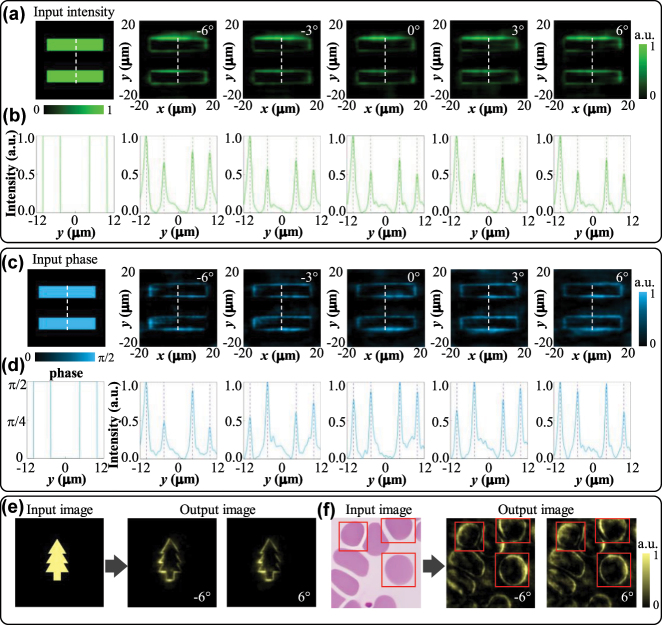
Schematics of the varifocal edge-enhanced imaging. (a) The intensity distribution of the incident amplitude image and the corresponding edge-enhanced images obtained by the metalens at different relative angles Δ*θ*. (b) Normalized intensity curves along the dotted lines in (a). (c) The phase distribution of the incident pure phase image and the corresponding edge-enhanced images obtained by the metalens at different relative angles Δ*θ*. (d) The phase curve and normalized intensity curves along the dotted lines in (c). (e) Edge-enhanced images of the tree-shaped pattern. (f) Edge-enhanced images of the cell microscopy image, the red solid-line boxes highlight the same corresponding cells in different image panels.

In the *y*-polarization state, the Moiré metalens exhibits vortex-focusing phase distribution. Thus, the metalens enables isotropic edge-enhanced imaging of the input image based on the radial Hilbert transform [59]. Compared with brightfield imaging, the edge-enhanced imaging enables higher sensitive detection of objects with a large difference in the refractive index from the background. To test the edge-enhanced imaging capability of the Moiré metalens, we choose the resolution-chart-like panel as the incident image, as illustrated in Figure 8a and c. We characterize both the amplitude and phase images to assess the edge-enhanced imaging performance. As for the incident amplitude image, the phase remains constant (*φ* = 0) and the input image is encoded by binary pixel intensities (0 or 1). The incident pure phase image exhibits a uniform intensity distribution (*I* = 1), with its light field expressed as $u_{0}=\exp\left(i\varphi \right)$ , where the phase *φ* is binary-encoded by pixels with *φ* = 0 or *π*/2. The edge-enhanced images of the varifocal Moiré metalens for the amplitude and phase objects are shown in Figure 8a and c, respectively. With the relative angle changing from −6° to 6°, the metalens can achieve varifocal edge-enhanced imaging from 56.4 μm to 64.7 μm. Figure 8b and d demonstrates the normalized intensity of the edge-enhanced images in the *y* direction. It’s illustrated that the peaks of output images match well with the incident image. The resolution of the metalens can be extracted from the FWHMs of the edge response. For the amplitude object, the resolution shifts from 1.84 μm to 1.6 μm, within the 12° range of rotation. For the phase object, the resolution changes from 1.95 μm to 1.3 μm within 12° angular variation. Moreover, by employing the tree-shaped pattern and the cell microscopy image as the incident images, we further tested the edge-enhanced capability of the Moiré metalens. As illustrated in Figure 8e and f, the proposed metalens maintains high-quality edge-enhanced imaging even for complex and irregular input images. The designed Moiré metalens proves practically applicable and shows potential in biomedical imaging and related fields.

## 4 Conclusions

In this work, we proposed a polarization-multiplexed varifocal Moiré metalens for QPI and edge-enhanced imaging. The metalens exhibits the Moiré phase profiles and the vortex phase profiles, respectively, in the *x*- and *y*-polarization states. Under *x*-polarization light incidence, the continuous zoom capability enables the metalens to capture multiple intensity profiles from 58.7 μm to 61.8 μm. Combined with AS-TIE method, the metalens achieves high-precision phase retrieval with an RMSE of 0.015 rad. Furthermore, the metalens can achieve a dynamic switch from QPI to edge-enhanced imaging by changing the incident polarization state. Under *y*-polarization light incidence, the varifocal edge-enhanced imaging can be realized for both the amplitude and phase objects with the minimum spatial resolution reaching 1.3 μm. QPI is noninterference phase imaging method suitable for unstained sample detection and observation. The depth and refractive index information can be derived from the reconstructed phase. Moreover, the edge-enhanced imaging is suitable for the edge positions with sharp or abrupt phase distributions, which are relatively difficult for QPI. The designed dual-mode varifocal Moiré metalens exhibits substantial potential for applications in microscopy, biomedical imaging, and optical metrology.
